# HIF-α activation by the prolyl hydroxylase inhibitor roxadustat suppresses chemoresistant glioblastoma growth by inducing ferroptosis

**DOI:** 10.1038/s41419-022-05304-8

**Published:** 2022-10-08

**Authors:** Xiaodong Su, Yuan Xie, Junwen Zhang, Mingxin Li, Qing Zhang, Guishan Jin, Fusheng Liu

**Affiliations:** 1 Brain Tumor Research Center, Beijing Neurosurgical Institute, Capital Medical University, Beijing, 100070 China; 2 Department of Neurosurgery, Beijing Tiantan Hospital Affiliated to Capital Medical University, Beijing, 100070 China; 3 Beijing Laboratory of Biomedical Materials, Beijing, 100070 China

**Keywords:** CNS cancer, Cell death

## Abstract

Patients with glioblastoma (GBM) have poor prognosis and limited treatment options, largely due to therapy resistance upon the induction of apoptosis. Ferroptosis emerges as a potential antineoplastic strategy to bypass apoptosis resistance in traditional therapeutics. Hypoxia is a fundamental hallmark of GBM and hypoxia-inducible factor (HIF) is the main regulator of hypoxia response, however, the role of HIF has not been sufficiently explored in GBM. Herein, we first discovered that amplifying HIF signals by the prolyl hydroxylase (PHD) inhibitor roxadustat significantly suppressed GBM cell growth in vitro and in vivo, especially when the cells were resistant to temozolomide (TMZ). The accumulation of lipid peroxidation and cellular iron in GBM cells following roxadustat treatment indicated that the cells underwent ferroptosis, which was also supported by morphological changes in mitochondrial ultrastructure and immunogenic signals release. Moreover, in vivo studies further confirmed the ferroptosis induction and verified that roxadustat significantly prolonged survival of the mice harboring chemoresistant GBM without visible organ toxicity. Finally, we proved that the ferroptosis induction by roxadustat is HIF-α independent, especially activation of HIF-2α upregulating lipid regulatory genes was revealed to be mainly responsible for the enhanced lipid peroxidation. Altogether, our study provided novel evidence that amplifying HIF signals induced ferroptosis in chemoresistant GBM cells and suppressed the tumor growth in vivo, highlighting that ferroptosis induction by targeting HIF-α might provide new approaches to improve GBM treatment.

## Introduction

Glioblastoma (GBM) is the most common and aggressive primary brain tumor in adults and accounts for more than 30% of intracranial tumors [1, 2]. Most patients with GBM have an expected survival of less than 2 years despite standard multimodal intervention consisted in maximal resection followed by radiotherapy and chemotherapy with the alkylating agent temozolomide (TMZ) [3]. It has been found that GBM cells easily develop resistance to apoptosis, which confers tumor recurrence and treatment failure [4]. Novel therapeutic strategies are needed to improve patient outcome.

Ferroptosis is a newly identified form of regulated cell death. Compared with apoptosis, ferroptosis is iron-dependent and characterized by the accumulation of free iron and lipid peroxidation that leads to cell death [5, 6]. Particularly, cancer cells resistant to conventional therapies, including those with strong mesenchymal and metastatic properties, are found more susceptible to ferroptosis [7–9]. Activating ferroptosis has emerged as a potent mechanism for targeting cancer cells. GBM cells exhibit increased iron uptake and high free iron levels, implying their intrinsic predisposition to ferroptosis [10, 11]. Moreover, recent studies have shown that in GBM promoting ferroptosis resulted in decreased tumor growth and treatment resistance [12]. These findings suggest that ferroptosis induction may be a potential modality, aiding the current post-operative treatments for GBM.

Currently, system xc- and GPX4 inhibition are the most common methods to induce ferroptosis in cancer cells [13]. However, these methods haven’t proven applicable in GBM. Sulfasalazine, an oral anti-inflammatory drug, inhibits system xc- to induce ferroptosis and was found to lack efficacy and provoke serious neurological adverse events in clinical trials with malignant gliomas patients [14]. Further studies proved that inhibition of system xc- inhibits not only the influx of cysteine but also the efflux of glutamate, a molecule involved in the initiation of seizures and neuropathic pain [15, 16]. As for GPX4 inhibition, potential adverse side effects related to GPX4 targeting is thought to be challenges for clinical use, since GPX4 knockout causes embryonic lethality in mice [17]. Therefore, it is urgently needed to develop novel targets for ferroptosis induction in GBM.

Some oncogenic pathways are increasingly recognized to regulate ferroptosis in cancer cells [5, 8]. Hypoxia is a fundamental hallmark of GBMs and has been shown to be highly associated with tumor invasion and therapeutic response [18]. Hypoxic responses are transcriptionally controlled by hypoxia-inducible factors (HIFs), which are heterodimers comprising of an α-subunit (HIF-1α, HIF-2α or HIF-3α) and a β-subunit (HIF-β). HIF-1α and HIF-2α are considered the main regulators of hypoxia and positively associated with the malignant progression of various tumors [19, 20]. However, the effect of HIF-1α in GBM is controversial and HIF-1α is significantly correlated with IDH1/2 mutation [21]. Moreover, HIF-2α has been thought to have similar effect to HIF-1α and less reported in GBM. The role of HIF pathway in regulating ferroptosis in GBM has remained unclear.

Roxadustat is a small-molecule stabilizer of HIF by inhibiting prolyl hydroxylase (PHD) that hydroxylates HIF-α for degradation, and it’s approved as a first-in-class orally active drug for the treatment of renal anemia [22]. In this study, we use roxadustat to amplify HIF pathway in GBM cells and found that roxadustat significantly inhibited GBM cell growth in vitro and in vivo by inducing ferroptosis, especially when the cells were TMZ-resistant. Besides, roxadustat prolonged survival of the mice harboring chemoresistant GBM without causing obvious organ toxicity. Moreover, we demonstrated that activation of HIF-1α and HIF-2α both contributed to the ferroptosis induction by roxadustat and the increased HIF-2α upregulating lipid genes was mainly responsible for the excessive lipid peroxidation during the ferroptosis. Taken together, our findings provide new insights into the role of HIF signals in ferroptosis regulation and ferroptosis-based cancer therapy in GBM.

## Results

### Roxadustat inhibited GBM cell viability and induced TMZ-resistant GBM cell death

**Fig. 1:**
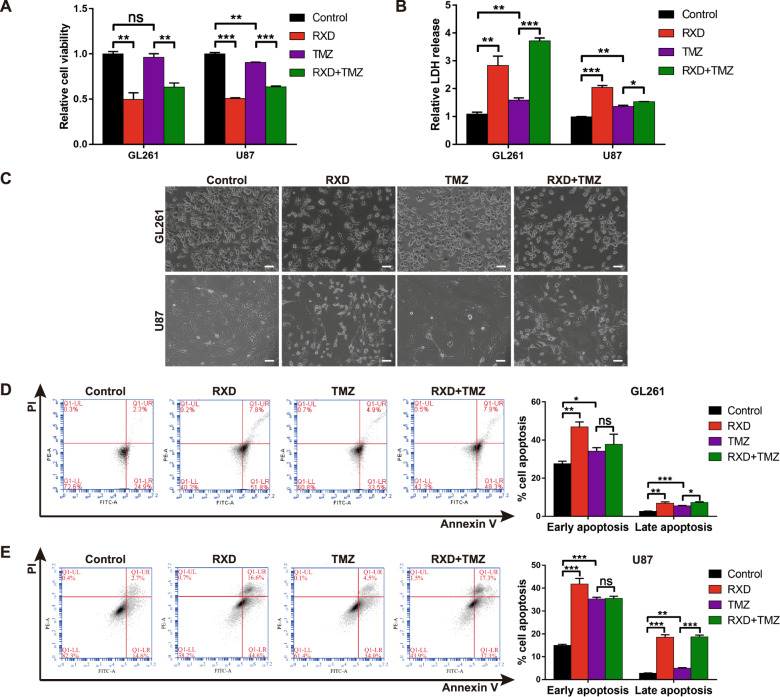
Roxadustat inhibited GBM cell viability and induced chemoresistant GBM cell death. **A** Cell viability assay in GL261 and U87 cells treated with RXD (100 μM towards U87, 200 μM towards GL261) for 48 h and/or TMZ (200 μM) for 48 h. **B** The LDH released in GL261 and U87 cells treated with RXD and/or TMZ for 72 h. **C** Representative images under the microscope of GL261 or U87 cells treated with RXD and/or TMZ for 48 h. Scale bar: 100 μm. **D** and **E** Apoptosis analysis of GL261 or U87 cells treated with RXD and/or TMZ for 72 h. *P < 0.05, **P < 0.01, ***P < 0.001, ns no significant difference.

We performed cell viability assay to investigate the effect of roxadustat (RXD) on both GL261 and U87 GBM cells. It was found that RXD significantly inhibited GBM cell viability, while the cells were quite resistant to TMZ (Fig. 1A). LDH release assay examined cell death (Fig. 1B) and revealed that RXD alone or combined with TMZ significantly induced more GBM cell death than TMZ alone. Consistently, the cell morphology also demonstrated RXD or combined with TMZ significantly decreased cell density and caused more cell roundness, compared with TMZ alone (Fig. 1C). These results indicated that RXD presented significant cytotoxicity towards these TMZ-resistant GBM cells, and there seemed to be no obvious synergistic interaction between RXD and TMZ.

We then investigated whether the cell death was caused by apoptosis, which is the main mechanism underlying antitumor effect of TMZ [23]. According to apoptosis analysis in GL261 and U87 cells, both RXD and TMZ increased cell apoptosis while RXD had a stronger effect on late apoptosis; On the other hand, the combination of RXD and TMZ didn’t induce more early apoptosis than TMZ alone (Fig. 1D, E), suggesting other mechanisms than apoptosis underlying the cytotoxicity of RXD. Moreover, we also examined the apoptosis in U251 cells that displayed less resistance to TMZ than U87 cells (Fig. S1A). Different from GL261 and U87 cells, U251 cells showed more TMZ-induced apoptosis and also more resistance to RXD, particularly, RXD combined with TMZ no longer enhanced the late apoptosis (Fig. S1B). These results suggested that RXD maintained potent cytotoxicity to GBM cells, especially towards those were chemoresistant.

### Roxadustat induced ferroptosis in chemoresistant GBM cells

**Fig. 2:**
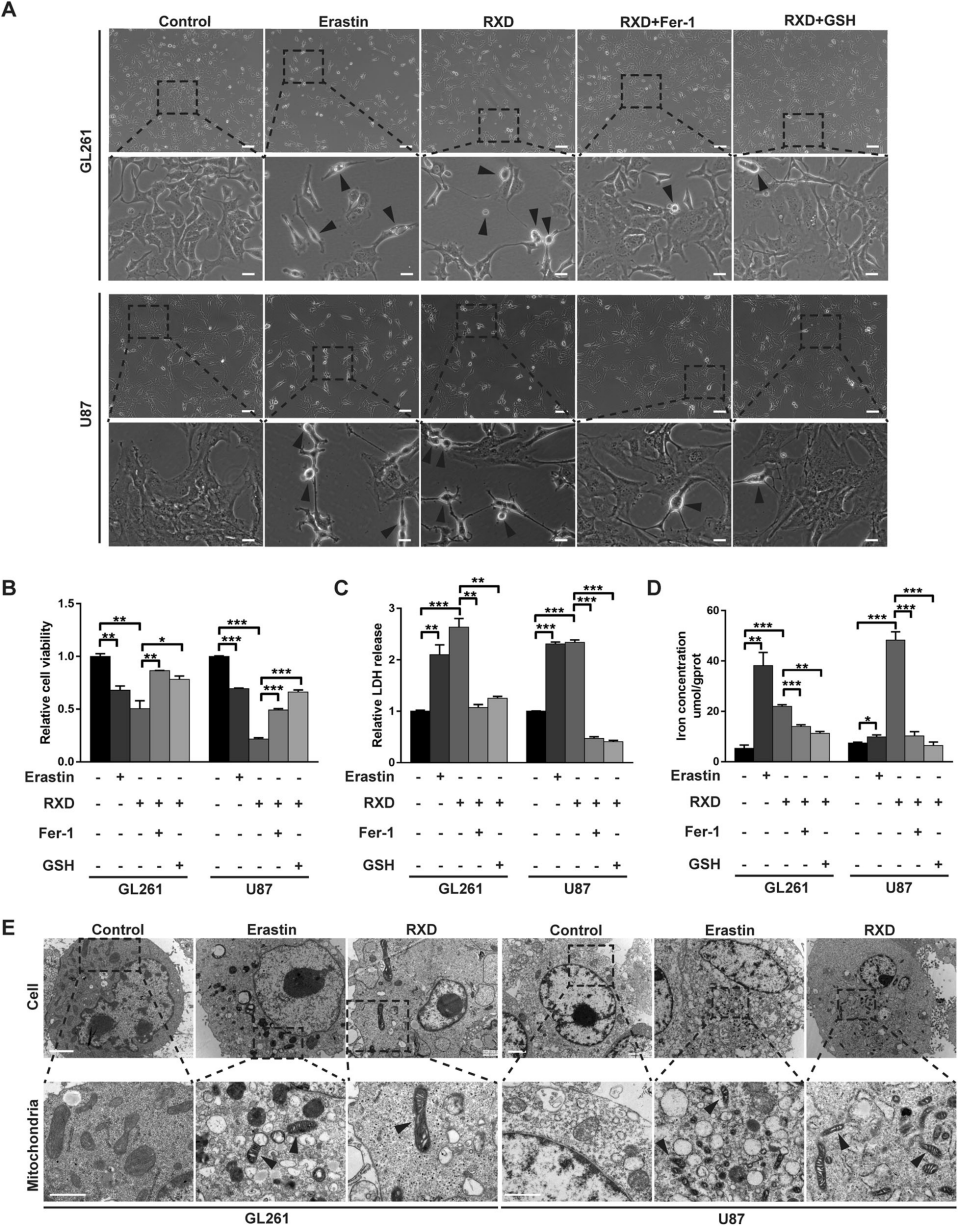
Roxadustat induced chemoresistant GBM cell death by causing ferroptosis. **A** Representative images of GL261 or U87 cells exposed to Erastin (10 μM), RXD (100 μM towards U87, 200 μM towards GL261) with or without the presence of Fer-1 (1 μM) or GSH (500 μM) for 48 h. Scale bar: 100 μm (up); 25 μm (down). **B** Cell viability assay of GL261 or U87 cells treated with different reagents for 48 h. **C** The LDH released in GL261 or U87 cells treated with different reagents for 72 h. **D** The cellular iron concentration in GL261 or U87 cells with different treatment for 48 h. **E** Representative cell and mitochondrial ultrastructural images of GL261 or U87 cells exposed to different reagents for 48 h. Scale bars: cell, 2 μm; mitochondria, 1 μm. *P < 0.05, **P < 0.01, ***P < 0.001.

To elucidate RXD-induced cell death, we used the ferroptosis inhibitors ferrostatin-1 (Fer-1) [7, 24] and glutathione (GSH) [7, 25] to pretreat U87 and GL261 GBM cells and the famous ferroptosis inducer Erastin was used as a positive control [7, 24, 25]. As is shown in Fig. 2A, obvious morphological alterations appeared in GBM cells following RXD or Erastin treatment, such as distinct cell swelling and decreased cell density, which were remarkably inhibited with the presence of Fer-1 or GSH. Also, the cell viability and LDH release assay consistently showed that RXD or Erastin significantly induced cell death in GBM cells and Fer-1 or GSH blocked the induction by RXD (Fig. 2B, C). Increased iron is an important hallmark of ferroptosis [10]. RXD or Erastin greatly elevated cellular iron concentration and Fer-1 or GSH restored these changes to the control level (Fig. 2D). Meanwhile, cells treated with RXD or Erastin displayed mitochondrial atrophy and increased mitochondrial membrane density, which represented morphological features of ferroptosis (Fig. 2E). All these results indicated the ferroptosis-inducing role of RXD in GBM cells.

**Fig. 3:**
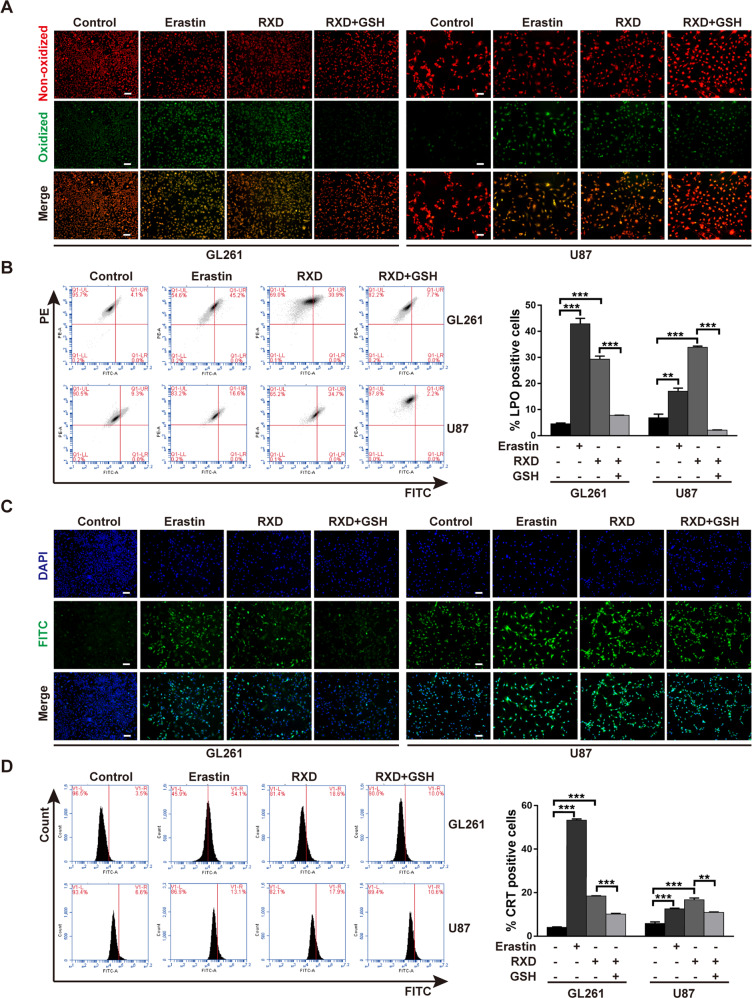
Roxadustat increased lipid peroxidation and calreticulin exposure. **A** Representative fluorescent images of lipid peroxidation in GL261 or U87 cells after incubation with Erastin (10 μM) or RXD (100 μM towards U87, 200 μM towards GL261) with or without GSH (500 μM) for 48 h. Scale bar: 100 μm. **B** Representative flow cytometry analysis and statistical histogram of LPO-positive cells in GL261 or U87 cells with the different treatments. **C** Representative fluorescent images of CRT in GL261 or U87 cells after the different treatments. Scale bar: 100 μm. **D** Representative flow cytometry analysis and statistical histogram of CRT-positive cells in GL261 or U87 cells after the different treatments. *P < 0.05, **P < 0.01, ***P < 0.001.

Increased lipid peroxidation (LPO) is another hallmark of ferroptosis [10], and we detected LPO by fluorescent probe BODIPY 581/591 C11. Erastin or RXD significantly increased oxidized lipid in both U87 and GL261 cells, while GSH inhibited this increase (Fig. 3A). Flow cytometry analysis confirmed the elevated proportion of LPO-positive cells by RXD and the neutralization effect of GSH (Fig. 3B). Moreover, ferroptosis is known as immunogenic cell death (ICD), characterized by releasing immunologic danger signals from dying cells [5, 26, 27]. As one of key danger-related molecular patterns, calreticulin (CRT) gets exposed on the cell surface early in the course of ICD [28]. Our results showed that the level of extracellular CRT in GL261 and U87 cells with RXD treatment was 4.6-fold and 2.8-fold of that in control, respectively, while GSH partially inhibited the increased CRT exposure (Fig. 3D). Immunofluorescent staining also showed increased CRT expression after Erastin or RXD treatment and the inhibition by GSH (Fig. 3C). Additionally, we also verified that combination of RXD and TMZ induced more LPO-positive and CRT-positive cells than TMZ alone (Fig. S1C-D), further demonstrated the difference between RXD- and TMZ-induced cell death. All together, these results confirmed that RXD induced GBM cell death by causing ferroptosis.

### Roxadustat suppressed chemoresistant GBM growth in vivo and prolonged survival of the GBM mice

**Fig. 4:**
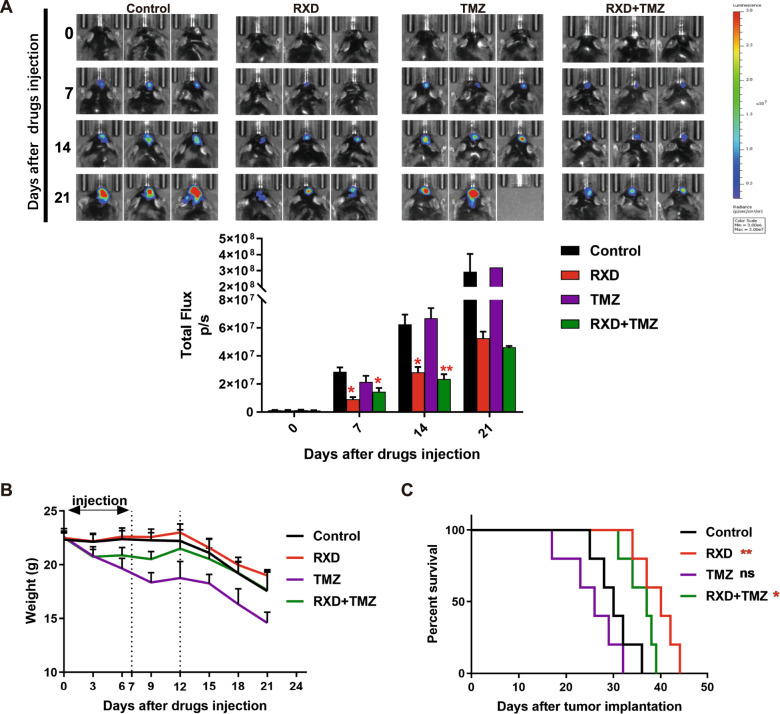
Roxadustat suppressed chemoresistant GBM growth in vivo and prolonged survival of the GBM mice. **A** IVIS images and associated ROI quantification of orthotopic GBM-bearing mice injected with RXD (10 mg/kg) and/or TMZ (35 mg/kg), measured every 7 days (0–21 days). *P < 0.05, **P < 0.01. **B** The body weight of mice in each group after drug injection was monitored and recorded every 3 days (0–21 days). **C** The survival curves of orthotopic GBM-bearing mice (*n* = 5). *P < 0.05, **P < 0.01.

To determine the antitumor effect of RXD in vivo, a mouse GBM orthotopic allograft model was established with GL261 cells. As shown in IVIS images and associated ROI quantification (Fig. 4A), TMZ monotherapy had no significant effect on the tumor growth, which further confirmed TMZ chemoresistance of GL261 cells; RXD monotherapy or the combination treatment significantly inhibited GBM growth after treatment initiation; No statistical analysis was performed on day 21 due to insufficient mice left in TMZ group, but tumor-growth trends in each group were consistent with in vitro studies. Survival analysis showed that the median survival of control, TMZ monotherapy, RXD monotherapy and combination treatment group was 30 days, 26 days, 40 days and 37 days, respectively (Fig. 4C). These results demonstrated that RXD maintained antitumor effect on the mice harboring chemoresistant GBM.

During drug treatment (day 0–7), weight loss occurred in both combination treatment and TMZ monotherapy group, but not in RXD monotherapy group. After drug injection, weight loss continued in TMZ monotherapy group, while it was significantly improved in the combination treatment group until gradual weight loss in all groups caused by growing tumor burdens (Fig. 4B). Also, we observed that the tumor-bearing mice had more active life status and glossier hairs in RXD group and combination treatment group than those in TMZ monotherapy, as recorded every 3 days (Supplementary video). These results implied that mice with TMZ treatment experienced severe side effects, which was attenuated, to some extent, by RXD treatment. Meanwhile, RXD appeared to be well-tolerated.

### Roxadustat induced ferroptosis in chemoresistant GBM tissues without causing significant visceral toxicity

**Fig. 5:**
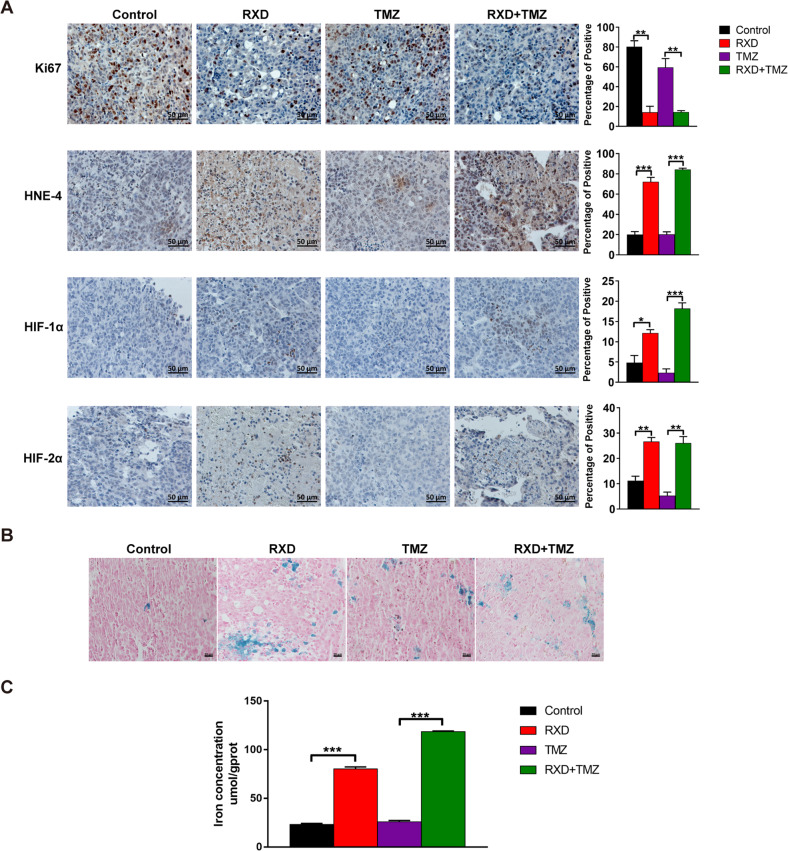
Roxadustat increased lipid peroxidation and iron concentration in vivo. **A** Representative immunohistochemical staining images and statistical histogram of Ki67, 4-HNE, HIF-1α and HIF-2α expression in GBM tissues from mice in each group. Scale bars: 50μm. **B** Representative Prussian blue staining images of GBM tissues from each group. Scale bars: 20 μm. **C** The iron concentrations of GBM tissues from mice in each group. *P < 0.05, **P < 0.01, ***P < 0.001.

Tumor tissues derived from the GBM mice were used to detect whether ferroptosis was induced by RXD in vivo. 4-Hydroxynonenal (4-HNE) is a by-product of lipid peroxidation and widely recognized as a stable marker of oxidative stress [7]. Through immunohistochemical staining, we found that fewer Ki67-positive cells and more 4-HNE-positive cells existed in GBM tissues from RXD or combination treatment group, illustrating that higher lipid peroxidation caused a decline in cell viability (Fig. 5A). Moreover, both HIF-1α-positive and HIF-2α-positive cells considerably increased in GBM tissues after RXD treatment, which was positively correlated with the level of lipid peroxidation (Fig. 5A). Prussian blue staining and iron concentration assay of GBM tissues consistently showed that RXD induced an increase of cellular iron and TMZ did not (Fig. 5B). Therefore, RXD increased cellular iron and lipid peroxidation inducing ferroptosis in vivo.

**Fig. 6:**
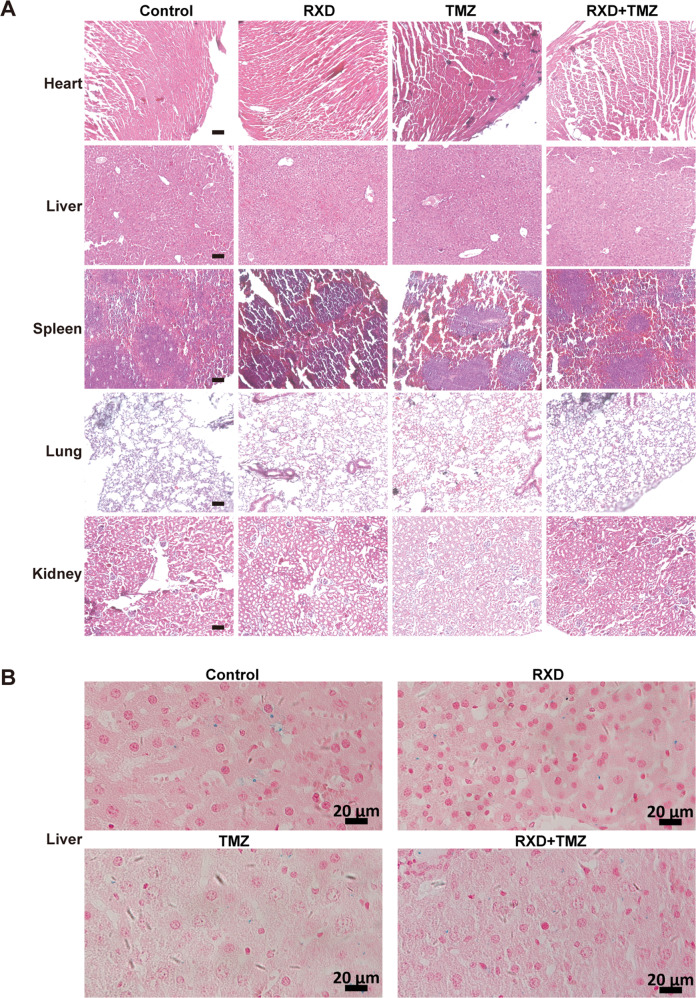
No significant visceral toxicity was observed at the end of observation. **A** Representative H&E staining images of heart, liver, spleen, lung, and kidney tissues from mice in each group. Scale bars: 100 μm. **B** Representative Prussian blue staining images of liver tissues from mice in each group. Scale bars: 20 μm.

In addition, pathomorphological analysis with heart, liver, spleen, lung, and kidney specimen from each group showed no visible pathological signs of toxicity in internal organs (Fig. 6A). Prussian blue staining revealed no obvious iron accumulation in livers of all treated mice (Fig. 6B). Therefore, RXD didn’t cause notable organ toxicity, suggesting good tolerance as an antitumor drug for GBM therapy.

### HIF-α activation mediated roxadustat-induced ferroptosis in chemoresistant GBM cells

**Fig. 7:**
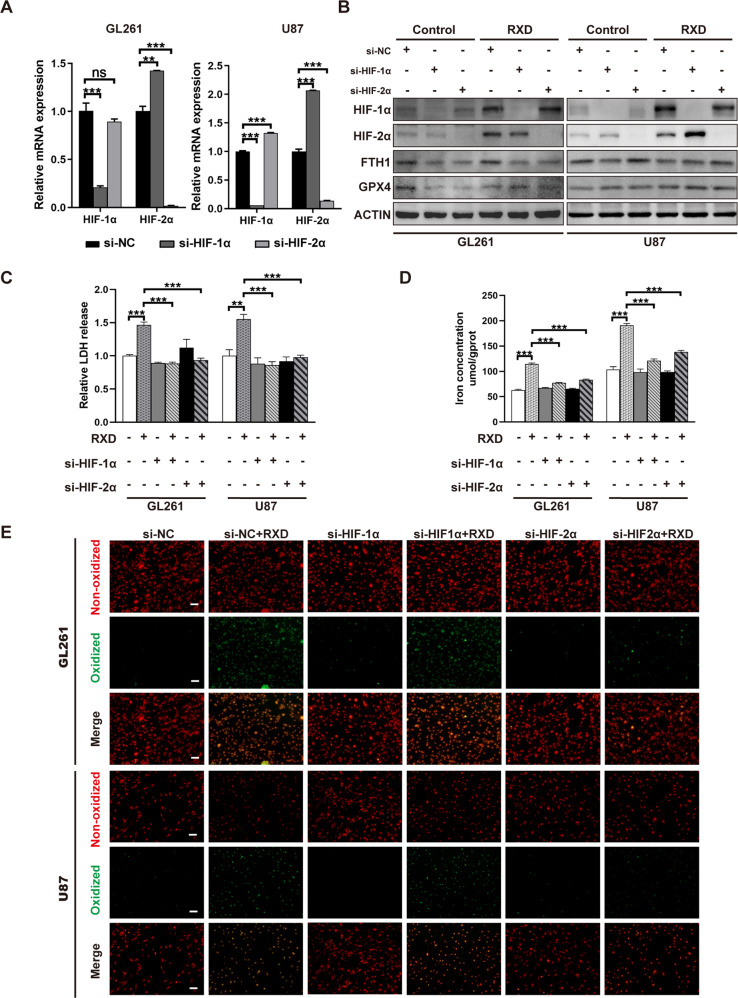
HIF-α activation mediated roxadustat-induced ferroptosis in chemoresistant GBM cells. **A** Histogram of mRNA expression of HIF-1α and HIF-2α in GL261 and U87 cells transfected with HIF-1α, HIF-2α and scrambled siRNAs for 48 h. **B** Western blotting analysis of protein expression in GL261 and U87 cells transfected with siRNAs for 48 h and with/without RXD treatment (100 μM towards U87, 200 μM towards GL261) for 48 h. **C** LDH release in GL261 or U87 cells transfected with siRNA for 48 h and treated with/without RXD for 72 h. **D** Cellular iron concentration of GL261 or U87 cells transfected with siRNA and treated with/without RXD for 48 h. **E** Representative fluorescent images of lipid peroxidation in GL261 or U87 cells in each group. Scale bar: 100 μm. *P < 0.05, **P < 0.01, ***P < 0.001.

**Fig. 8:**
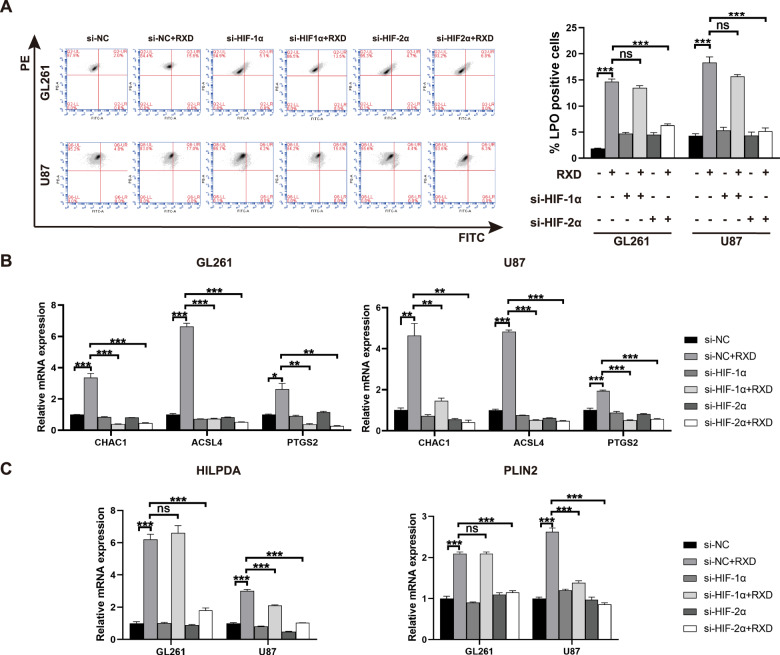
HIF-2α activation upregulating lipid genes contributed to excessive lipid peroxidation in roxadustat-induced ferroptosis. **A** Representative flow cytometry analysis and statistical histogram of LPO-positive cells in GL261 or U87 cells transfected with siRNAs for 48 h and with/without RXD treatment (100 μM towards U87, 200 μM towards GL261) for 48 h. **B** Histogram of mRNA expression of CHAC1, ACSL4, and PTGS2 in GL261 or U87 cells with the different treatments. **C** Histogram of mRNA expression of HILPDA and PLIN2 in GL261 or U87 cells with the different treatments. *P < 0.05, **P < 0.01, ***P < 0.001.

Roxadustat is a prolyl hydroxylase inhibitor that activates both HIF-1α and HIF-2α, then we studied the effect of HIF-α activation on RXD-induced ferroptosis in GBM cells. siRNA was used to interfere with the expression of HIF-1α or HIF-2α, both qPCR and western blotting analyses verified the effectiveness and specificity of the interference. Figure 7A, B showed that both HIF-1α and HIF-2α expression was significantly inhibited by siRNA in both GL261 and U87 cells. Besides, Fig. 7B also confirmed the activation of HIF-α by RXD. LDH release assay showed that knockdown of HIF-1α or HIF-2α both suppressed the cell death induced by RXD in GL261 and U87 cells (Fig. 7C). We further determined whether HIF-α activation affected lipid peroxidation and cellular iron in GL261 and U87 cells. Iron concentration assay showed that knockdown of HIF-1α or HIF-2α both could significantly block RXD-induced increase of cellular iron in the GBM cells (Fig. 7D). The Green fluorescence analysis with fluorescence microscopy and flow cytometry both confirmed that knockdown of HIF-2α rather than HIF-1α significantly inhibited the increased oxidized lipid caused by RXD in both GBM cells (Figs. 7E and 8A). Altogether, these results suggested that both HIF-1α and HIF-2α contributed the ferroptosis induction by RXD and the HIF-2α activation may mainly regulate lipid peroxidation during the ferroptosis in chemoresistant GBM cells.

### HIF-2α activation upregulating lipid genes contributed to excessive lipid peroxidation during roxadustat-induced ferroptosis

To further study the underlying mechanism of HIF-α-mediated ferroptosis in GBM cells, ferroptosis-related genes/proteins were detected. Western blotting showed that RXD, as a stabilizer of HIF, elevated expression of both HIF-1α and HIF-2α, and the siRNA transfection effectively blocked the upregulation (Fig. 7B). GPX4 and FTH1 are two classical protein markers also protective factors in ferroptosis [29, 30]. No significant difference was shown in FTH1 expression between each sample, and GPX4 expression following the different treatments didn’t significantly changed (Fig. 7B), suggesting that HIF-α-mediated ferroptosis involved other pathways bypassing GPX4 and FTH1. The overexpression of CHAC1 [29], ACSL4 [31] and PTGS2 [30], has been considered as a biomarker of ferroptosis. We showed that the expression of CHAC1, ACSL4 and PTGS2 was significantly upregulated by RXD and this upregulation was greatly blocked by the knockdown of HIF-1α or HIF-2α (Fig. 8B), further confirming the role of HIF-α in RXD-induced ferroptosis. Moreover, we proved that two lipid regulatory genes HILPDA and PLIN2, important for ferroptosis sensitization in renal cancers, were significantly upregulated following RXD treatment and the upregulation was merely HIF-2α-dependent (Fig. 8C). These results were consistent with that of lipid peroxidation analyses (Figs. 7E and 8A), suggesting that RXD-induced HIF-2α activation upregulating lipid genes is mainly responsible for the enhanced lipid peroxidation during the ferroptosis induction.

## Discussion

Ferroptosis, a form of non-apoptotic cell death, has recently attracted great attention as a potential antineoplastic strategy to overcome apoptosis resistance. Emerging evidence has suggested that some oncogenic pathways are related to ferroptosis, rendering cancer cells remarkably vulnerable to ferroptotic death [32, 33]. The effect of HIF-mediated signals on ferroptosis in GBM cells, to our knowledge, has not been reported. In this study, we provide novel evidence that amplification of HIF signals by small molecule drug roxadustat induces ferroptosis in chemoresistant GBM cells in a HIF-α dependent manner.

Our data demonstrates that ferroptosis was the major mechanism of cell death induced by roxadustat. Increased lipid peroxidation and iron accumulation are two crucial events in ferroptosis and excessive iron triggers ferroptosis through producing ROS by Fenton reaction [34]. Firstly, roxadustat was found to significantly induce cell death and viability inhibition in U87 and GL261 GBM cells, while the cells exhibited obvious resistance to TMZ. Subsequently, the cell death was proved to be paralleled by increases of lipid peroxidation and cellular iron and blocked by ferroptosis inhibitors Fer-1 and GSH, presenting the characterisitics of ferroptosis. The roxadustat-induced cell death also displayed ferroptosis-associated morphological and immune features, including mitochondrial abnormalities and CRT exposure. The mouse models of orthotopic GBM with GL261 cells were used and verified the ferroptosis induction in vivo. Therefore, all these results indicated that the GBM cells after roxadustat treatment underwent ferroptosis. For now, there are very few studies in effect of roxadustat on GBM cells. A study in colon cancer cells reported that roxadustat treatment led to a ferroptosis-susceptible cell state and significantly potentiated ferroptosis induced by erastin and RSL3, whereas, roxadustat treatment alone had no effect on cancer cell growth [35]. These differences suggest that the effect of roxadustat on cancer cells maybe tumor type-dependent and GBM cells may especially have intrinsic vulnerability for ferroptosis induction.

Given the prevalence of TMZ chemoresistance in GBM cells, we compared the effect of roxadustat in three GBM cell lines including human U87, U251 and mouse GL261 cells, which displayed varying degrees of TMZ resistance. It is noteworthy that U87 and GL261 cells exhibited obvious resistance to TMZ-induced apoptosis in vitro and in vivo, while they were susceptible to roxadustat-induced ferroptosis. Conversely, the U251 cells presented less resistance to TMZ but more resistance to roxadustat (Supplement Fig. 1A, B). Therefore, the TMZ-resistant GBM cells seem to retained even more sensitivity to ferroptosis induction. These findings are consistent with the previous work on ferroptosis, which proved that drug-tolerant persister cells exhibit enhanced sensitivity to induction of ferroptosis [36–38]. What’s more, the mice with roxadustat treatment achieved significant survival benefits and no visible signs of intestinal injury were observed. All the results suggest that ferroptosis induction should be further considered to improve GBM treatment, especially for chemoresistant GBM, and inducing ferroptosis by roxadustat may provide a potential avenue to eliminate certain resistant and residual tumor cells in concert with other therapies in GBM.

Furthermore, our data suggests that roxadustat induces ferroptosis by HIF-α activation, especially HIF-2α activation was mainly responsible for the lipid peroxidation accumulation during the ferroptosis. Roxadustat is an inhibitor of PHD that degrades HIF-α and thereby augments HIF-α transcriptional activity in cells. We proved that the knockdown of HIF-1α or HIF-2α both significantly suppressed the GBM cell death (Fig. 7C) and increase of cellular iron (Fig. 7D) as well as inhibited upregulation of the hallmark genes (Fig. 8B), suggesting that both HIF-1α and HIF-2α activation contributed to the ferroptosis induction. Meanwhile, only the knockdown of HIF-2α rather than HIF-1α was shown to inhibited the roxadustat-induced increase of oxidized lipid (Figs. 7E and 8A) and overexpression of HILPDA and PLIN2, indicating HIF-2α as the major player during the ferroptosis induction. Our finding is consistent with the study in clear cell carcinoma where HIF-2α was shown to promote ferroptosis by upregulating lipid genes and stimulating the specific enrichment of polyunsaturated fatty acids [39], and these evidence supports the conclusion that HIF pathway is a key driver of ferroptosis vulnerability [33]. However, upregulations of lipid genes are not the whole story underlying the ferroptosis induction by HIF-α activation. The work done by Rashi et al. identified strong association of HIF-2α with ROS production and revealed that HIF-2α activation potentiated ROS, via an iron-dependent pathway and irreversible cysteine oxidation, and enhanced cell death in colorectal cancers [35]. Additionally, HIF-2α inactivates hepcidin and upregulates transferrin, ferroportin and divalent metal transporter-1 to promote iron absorption and transportation, thereby facilitating ferroptosis induction [22, 40]. Therefore, further investigations are needed to clarify all the underlying interactions between HIF-2α activation and ferroptosis induction in GBM cells.

Besides, it’s worth noting that the role of HIF-1α in the ferroptosis induction needs to be further clarified. The team of Donlin Tang found that HIF-1α stabilization promoted fatty acid uptake and lipid storage, then inhibited ferroptosis through elevated lipid droplet formation in human fibrosarcoma or lung cancer cells [41], suggesting the role of HIF-1α may vary as cell background. Especially, the pro-tumor effect of HIF-1α on malignant progression has been extensively reported [42, 43]. As one of the most famous targets of HIF1, VEGF gene is responsible for angiogenesis, which is known to result in poor patient prognosis. This somehow appears to be contradictory to the conclusion that HIF-α activation presents antitumor effect in GBM. How the role of HIF-α and hypoxia in angiogenesis will affect GBM prognosis after RXD treatment, as well as how to modulate the induction of angiogenesis and ferroptosis during the stabilization of HIFs to optimize GBM treatment, further systematic studies are needed to unveil these issues.

Roxadustat, also called FG-4592, was approved by the Chinese Government for the treatment of renal anemia in 2018 [44]. As a PHD inhibitor, roxadustat can stabilize HIF-2α then induce endogenous erythropoietin (EPO) production and improving erythropoiesis [44, 45], which may partially contribute to prolonged survival of mice with roxadustat treatment than those with TMZ monotherapy (Fig. 4C), since hematopoiesis is usually impaired during chemotherapy. Moreover, emerging evidence has revealed roxadustat to be promising for treating a variety of diseases beyond anemia [22]. Particularly, HIF-1α stabilization by roxadustat has been found to mitigate tissue injury. Li *et al*. reported that roxadustat pretreatment attenuated acute kidney injury by decreasing ferroptosis [46]. Besides, several studies have discovered the potential of roxadustat as a radioprotective agent in cancer therapy and indicated that it protected normal tissue from damage during radiotherapy via reducing cell apoptosis [47, 48]. It appears that pharmacological activation of HIF-α with roxadustat can protect tissue cells from injury through anti-ferroptosis while induce cancer cell death through pro-ferroptosis, which illustrates a considerable therapeutic window for optimizing cancer therapy. The role of HIF signals in ferroptosis seems to be cell background-dependent, suggesting that HIF activation by roxadustat may have the potential being included in personalized treatment.

In summary, we have shown for the first time that HIF-α activation by roxadustat induces ferroptosis in TMZ-resistant GBM cells, which significantly inhibits the GBM cell growth in vitro and in vivo without notable visceral toxicities. Our study has suggested that HIF-α, especially HIF-2α, is a promising therapeutic target for ferroptosis induction in chemoresistant GBM and exposed the potential of roxadustat as a new treatment option for patients with refractory GBM.

## Materials and methods

### Reagents and antibodies

Roxadustat (S1007), erastin (S7242), ferrostatin-1 (S7243), glutathione (S4606) and temozolomide (S1237) were from Selleck Chemicals (Texas, USA). BODIPY™ 581/591 C11 (D3861) was from Invitrogen, ThermoFisher (California, USA). Prussian Blue Iron Stain Kit (G1422) was from Solarbio Life Sciences (Beijing, China). Fluorescence labeled anti-Calreticulin (#62304) was from Cell Signaling Technology (CST). Primary antibodies were as follows: GPX4 (sc-166570, Santa Cruz), FTH1 (#3998 S, CST), HIF-1α (#36169 S, CST), β-actin (#4970 S, CST), HIF-2α (ab109616, abcam), 4-HNE (ab48506, abcam), Ki67 (sc-15402, Santa Cruz).

### Cell line and culture

GBM cell lines U87, U251 and GL261 were purchased from The Cell Center of the Chinese Academy of Medical Sciences (Beijing, China) and tested for mycoplasma contamination by Mycoplasma.Detection Kit (40612ES25, Yeasen, Shanghai, China). All the cells were cultured in Dulbecco’s modified Eagle’s medium with 4.5 g/L glucose (H-DMEM, Gibco, USA) containing 10% FBS (Gibco, USA), 100 U/ml penicillin and 100 μg/ml streptomycin (Gibco, USA) at 37 °C with 5% CO_2_.

### Animal experiments

All animal experiments were conducted in accordance with the Guide for the Care and Use of Animals. C57BL/6 N male mice (6-8 weeks) were purchased from Beijing Vital River Laboratory (Beijing, China). All mice were maintained under SPF conditions and at constant temperature (25 °C) and relative humidity (65%) with a 12 h light/dark cycle. Orthotopic implantation of GBM cells was performed as described previously [49]. In brief, mice were anesthetized with isoflurane and placed into a stereotactic apparatus (KOPF, USA). 1 × 10^5^ GL261-Luc cells were injected into the right striatum at a position 2 mm lateral and 1 mm anterior to the bregma and 3 mm below the brain surface.

On day 7 after implantation, mice were imaged to assess tumor growth using an IVIS platform (Xenogen IVIS Spectrum, Caliper LifeSciences, USA) and tumor-free mice were excluded. Mice were randomly allocated to 4 different groups (n = 10/groups): (1) control group that received an intraperitoneal (i.p.) injection of solvent vehicle (5% DMSO in ddH2O); (2) Roxadustat (RXD) group that received 10 mg/kg Roxadustat i.p. for 7 days (dissolved in DMSO at 50 mg/ml and further diluted in 5% DMSO with 40% PEG300, 5% Tween 80 and 50% ddH2O to 2.5 mg/ml); (3) Temozolomide (TMZ) group that received 35.7 mg/kg TMZ i.p. for 7 days (the dose based on human standard doses of 200 mg/m^2^ that converted for a mouse, dissolved in DMSO at 35.7 mg/ml and further diluted in 5% DMSO with 30% PEG300 and 65% ddH2O to 1.8 mg/ml); (4) RXD + TMZ group that received the RXD i.p. 6 hours before the TMZ i.p. for 7 days.

In vivo tumor growth images were obtained by the IVIS spectrum on day 0, 7, 14, 21 after the first injection. Animal weights were measured every three days until 21 days. Life status of mice was recorded by video every 3 days until end point. 3 to 4 mice in Each group were sacrificed on day 15 to harvest their tumor tissues and liver, lung, spleen, kidney, heart specimens were harvest at end points. The tissues were fixed with 4% PFA or frozen at −80 °C. Sections were prepared from fixed paraffin-embedded tissues, followed by hematoxylin-eosin (H&E), Perl’s Prussian blue stain, Ki67 (1:50), 4-HNE (1:25), HIF-1α (1:100), HIF-2α (1:200) staining. Frozen tissues were treated for iron analysis. Each group had at least 5 mice for survival analysis.

### Cell viability assay

Cell viability was analyzed with the Cell Counting Kit-8 (CCK-8 Kit, Dojindo Laboratories, Japan) according to the manufacturer’s protocol. Briefly, 2000 cells were seeded in 96-well plates per well and treated with different chemical regents for 48–72 hours. Then cells were incubated with CCK-8 for 2 h at 37 °C. The absorbance of each well was obtained by a microplate reader (PerkinElmer, USA) at 450 nm. At least four wells were used for each sample.

### Cell death assay

Cell death was evaluated by using lactate dehydrogenase (LDH) cytotoxicity assay kit (Beyotime Biotechnology, Nanjing, China). 2 × 10^3^ cells were seeded in 96-well plates per well and treated with chemical regents for 72 h, then collected to detect the absorbance of each sample at 490 nm according to the manufacturer’s instructions. Cell death ratio was calculated using the following formula: Cell death (%) = (Absorbance_sample_ − Absorbance_control_) / (Absorbance_maximum_ − Absorbance_control_) × 100, where Absorbance_maximum_ is the absorbance of the positive group. At least four wells were used for each sample.

### Apoptosis analysis

Apoptosis was detected with Annexin V-FITC/PI Apoptosis Detection Kit (Yeasen, Shanghai, China). 1 × 10^5^ cells were plated in the 12-well plate and treated with different compounds. After 72 h treatment, cells were harvested and incubated with FITC-conjugated Annexin V and PI according to the manufacturer’s instruction. Apoptotic cells were counted via flow cytometry on Accuri C6 flow cytometers (BD Biosciences, USA) and calculated by CFlow Plus software (BD Biosciences, USA). A total of at least 1 × 10^4^ cells was analyzed for each sample. All samples were tested in triplicate.

### Transmission electron microscopy

Cells were plated in 6-well plates (2 × 10^5^ cells/well) and treated with drugs for 48 h. Cells were digested and fixed with 2.5% glutaraldehyde in 2% PFA for 2 h at 4 °C. The samples were then post-fixed with 1% osmium tetroxide in PBS for 2 h, dehydrated through a graded ethanol series, embedded in EPON 812 and cut into ultrathin sections. The sections were stained with 1% uranyl acetate and 0.4% lead citrate before images of cellular ultrastructural morphology was obtained by TEM (H7650, Hitachi, Japan).

### Lipid ROS measurement

Cells were plated in 12-well dishes (20,000 cells/well) and treated with different drugs for 48 h. The cells were subsequently stained with C11-BODIPY 581/591 (10 μM) for 30 min at 37 °C according to the manufacturer’s instruction. After washes in PBS, images were taken with a fluorescence microscope (ZEISS Axio Observer A1, Germany). The cells were harvested and analyzed via flow cytometry to detect oxidized forms of the probe. Each sample included at least 10,000 cells for analysis and all samples were tested in triplicate.

### Iron concentration measurement

Iron content in cell lysates and tissues was determined with Serum Iron Assay Kit (A039-1) and Tissue Iron Assay Kit (A039-2) from Nanjing Jiancheng Bioengineering Institute (Nanjing, China), respectively. According to manufacturer’s protocol, cells (plated in 6-well plate with 2 × 10^5^ cells/well) and tumor tissues (10 mg) were collected and disrupted. The iron concentration was assessed by reading the absorbance of each sample at 520 nm and at least four wells were used for each sample.

### CRT assay

Cells were seeded in 24-well plates (20,000 cells/well) with 13-mm round glass coverslips and treated with drugs for 48 h. After fixed in 4% PFA and incubated with anti-CRT antibodies (1:50) overnight at 4 °C, the cells on coverslips were washed with PBS then stained with DAPI (Invitrogen, ThermoFisher, USA) and images were taken by a fluorescence microscope (ZEISS Axio Imager M2, Germany). For flow cytometer analysis, the drug-treated cells were digested and incubated with anti-CRT antibodies (1:50) for 30 min at 4 °C. Each sample included at least 10,000 cells for analysis and was tested in triplicate.

### Transfection of small interfering RNA

siRNA transfection used Lipofectamine 3000 (L3000015, Invitrogen, USA) with reduced serum medium Opti-MEM (319085062, Gibco, USA) according to the manufacturer’s instructions. siRNAs were designed and synthesized by GenePharma (Shanghai, China):

5’-GCCGCUCAAUUUAUGAAUATT-3’ (siRNA HIF-1α-homo);

5’-GCUGAUUUGUGAACCCAUUTT-3’ (siRNA Hif-1α-mouse);

5’-GGUGGAGCUAACAGGACAUTT-3’ (siRNA HIF-2α-homo);

5’-CCGACCAGCAAAUGGAUAATT-3’ (siRNA Hif-2α-mouse).

### Quantitative RT-PCR

Total RNA was extracted using TRIzol reagent (Invitrogen, USA) according to manufacturer’s instructions. cDNA synthesis was performed using Reverse Transcription System Kit (Promega A3500, USA). The PCR amplification was carried out in triplicates with SYBR premix Ex Taq (Takara, Japan) on a QuantStudio 6 Flex system (Applied Biosystems). Primers were synthesized by Sangon Biotech (Shanghai, China) and the sequences are listed in Supplementary Table S1. Relative mRNA expression was evaluated by the 2^–ΔΔCt^ method and normalized to ACTB expression.

### Western blot analysis

Total protein was extracted by RIPA lysis buffer with 1 mM PMSF proteinase inhibitors cocktail (Beyotime, China) and quantified using Pierce BCA Protein Assay kit (Thermo Scientific, USA). Protein extracts (15 μg) were separated by electrophoresis through 10% SDS-PAGE and then electroblotted onto 0.22 μm PVDF membranes (Millipore, Germany) and blocked in 5% milk in Tris-buffered saline containing Tween-20 (TBST) for 1 h at room temperature. The membranes were incubated with primary antibodies, including anti-GPX4 (1:1000), anti-FTH1 (1:1000), anti-HIF-1α (1:1000), anti-HIF-2α (1:1000) and anti-β-actin (1:5000) overnight at 4 °C, and HRP-conjugated secondary antibodies (Neobioscience, China) for 1 h at room temperature. Protein signals were detected with an enhanced chemiluminescent kit (Thermo Scientific, USA).

### Statistical analysis

Data were analyzed with GraphPad Prism 7 software and are presented as the means ± SD. Comparisons were performed using unpaired Student’s t-test between two groups and one-way analysis of variance (ANOVA) among three or more groups. Differences were considered statistically significant at *P < 0.05, **P ≤ 0.01 and ***P ≤ 0.001. ‘ns’ stands for *P* ≥ 0.05.

## Supplementary information

Figure S1

Table S1

photoes of Raw WB

checklist

Video of life status_control

Video of life status_RXD

Video of life status_RXD+TMZ

Video of life status_TMZ

## Data Availability

All datasets generated and analysed during this study are included in this published article and its Supplementary Information files. Additional data are available from the corresponding author on reasonable request.
