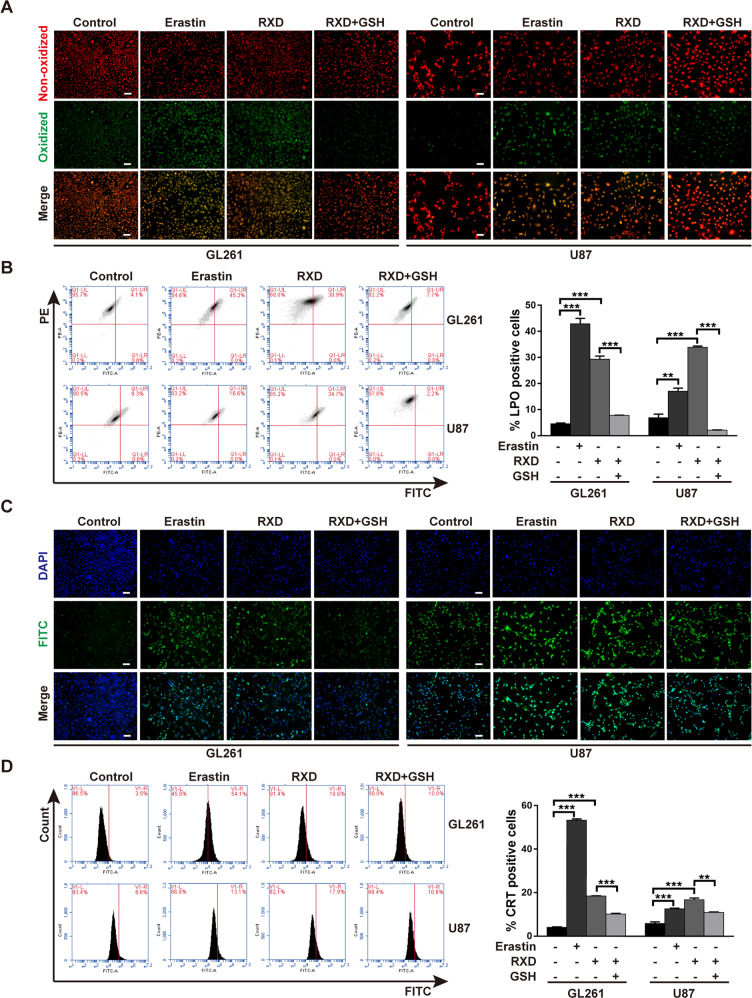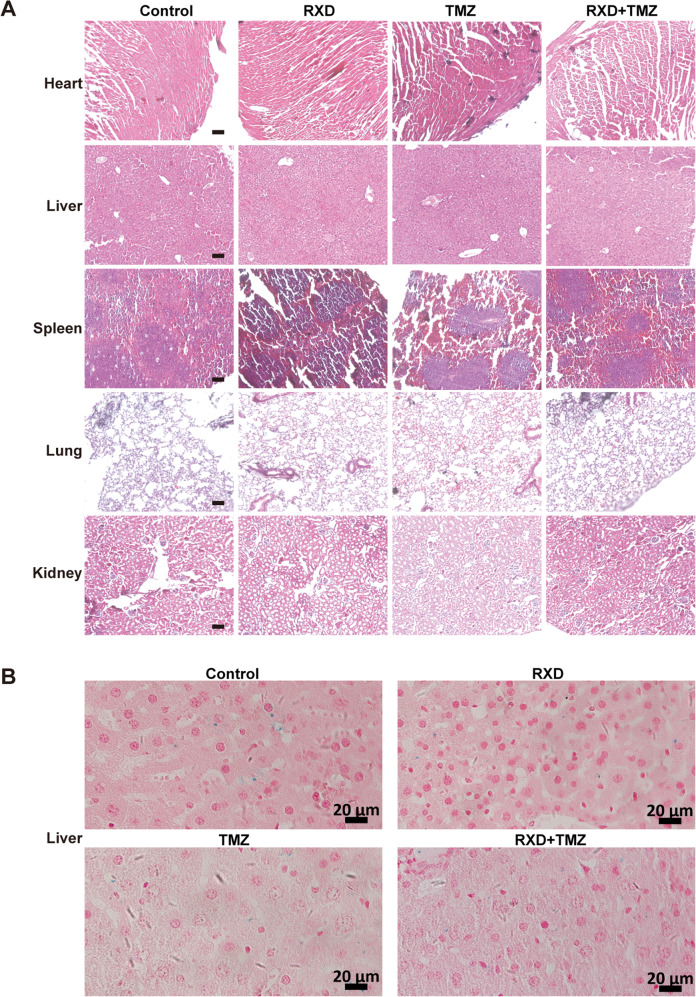# Correction: HIF-α activation by the prolyl hydroxylase inhibitor roxadustat suppresses chemoresistant glioblastoma growth by inducing ferroptosis

**DOI:** 10.1038/s41419-022-05532-y

**Published:** 2023-01-17

**Authors:** Xiaodong Su, Yuan Xie, Junwen Zhang, Mingxin Li, Qing Zhang, Guishan Jin, Fusheng Liu

**Affiliations:** 1 Brain Tumor Research Center, Beijing Neurosurgical Institute, Capital Medical University, Beijing, 100070 China; 2 Department of Neurosurgery, Beijing Tiantan Hospital Affiliated to Capital Medical University, Beijing, 100070 China; 3 Beijing Laboratory of Biomedical Materials, Beijing, 100070 China

**Keywords:** CNS cancer, Cell death

Correction to: *Cell Death and Disease* 10.1038/s41419-022-05304-8, published online 08 October 2022

The original version of this article contained two errors in Fig. 3C and Fig. 6B: in Fig. 3C, the merged image of the control GL261 cells was inadvertently duplicated and presented as both the DAPI and Merge images; in Fig. 6B, the liver image of the RXD+TMZ group was inadvertently duplicated and presented as both RXD and RXD+TMZ group livers. The source data files were not affected by the errors, and contain the correct images for all the groups. The authors apologize for the errors. The errors have been corrected in both the PDF and HTML versions of the Article.