# Supplementary figures and images for: HIF-α activation by the prolyl hydroxylase inhibitor roxadustat suppresses chemoresistant glioblastoma growth by inducing ferroptosis
Source: Cell Death Dis. 2022 Oct 8;13(10):861. doi: 10.1038/s41419-022-05304-8 (PMC9547873; doi:10.1038/s41419-022-05304-8)

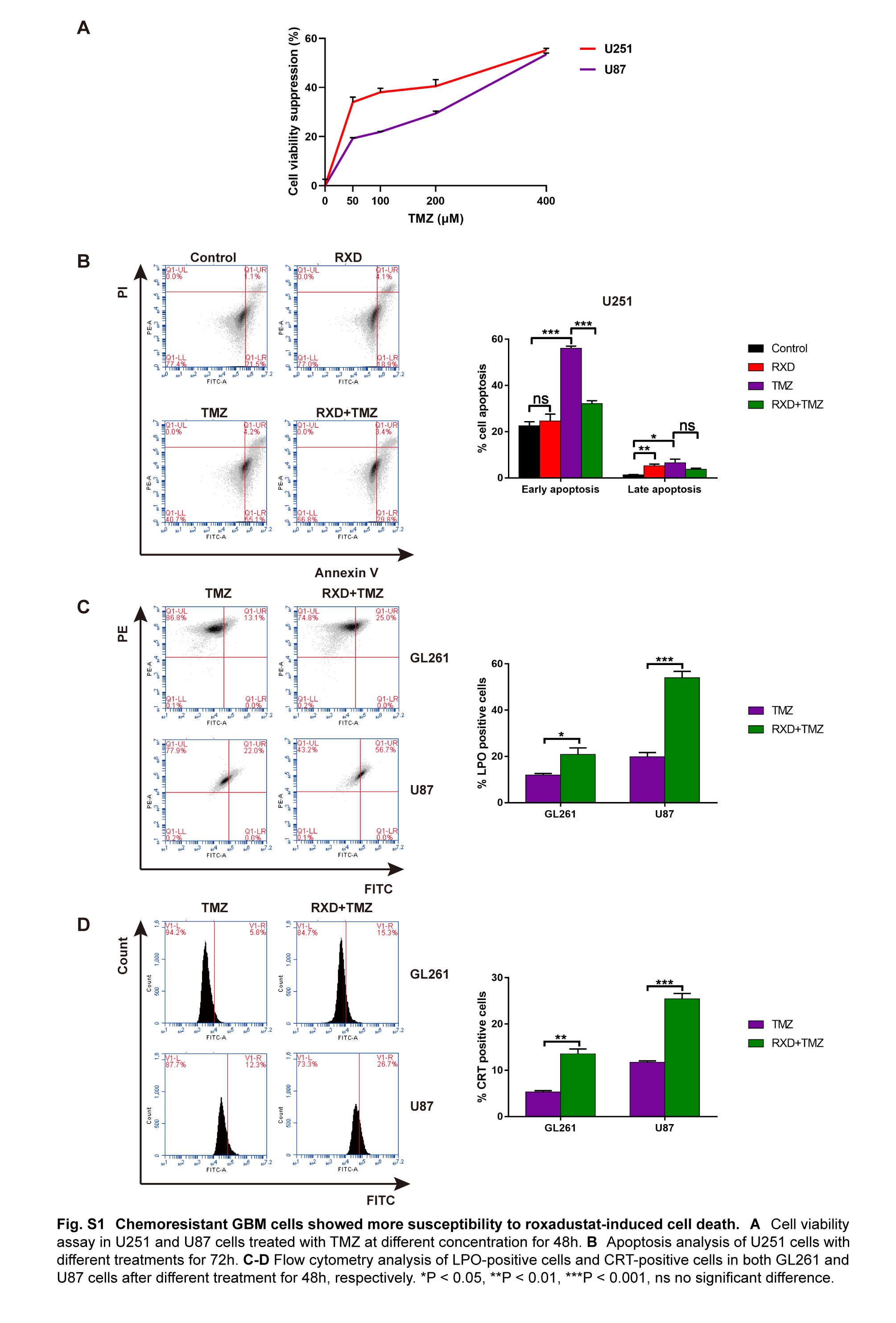

Supplement: Supplementary file 1 — Figure S1 [file 41419_2022_5304_MOESM1_ESM.tif]
