# Supplementary material for: HIF-α activation by the prolyl hydroxylase inhibitor roxadustat suppresses chemoresistant glioblastoma growth by inducing ferroptosis
Source: Cell Death Dis. 2022 Oct 8;13(10):861. doi: 10.1038/s41419-022-05304-8 (PMC9547873; doi:10.1038/s41419-022-05304-8)

Full length western blots for results in Figure 7B:

Fig.7B GL261-Actin


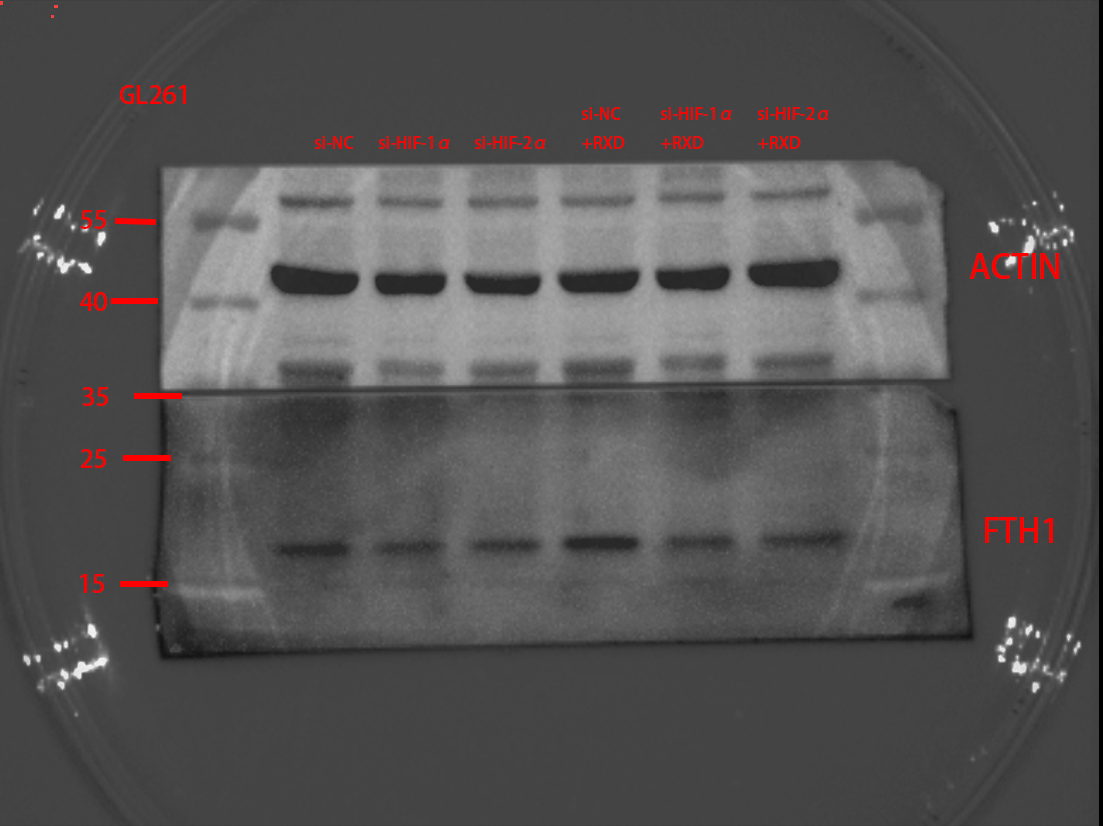


Fig.7B GL261-Fth1


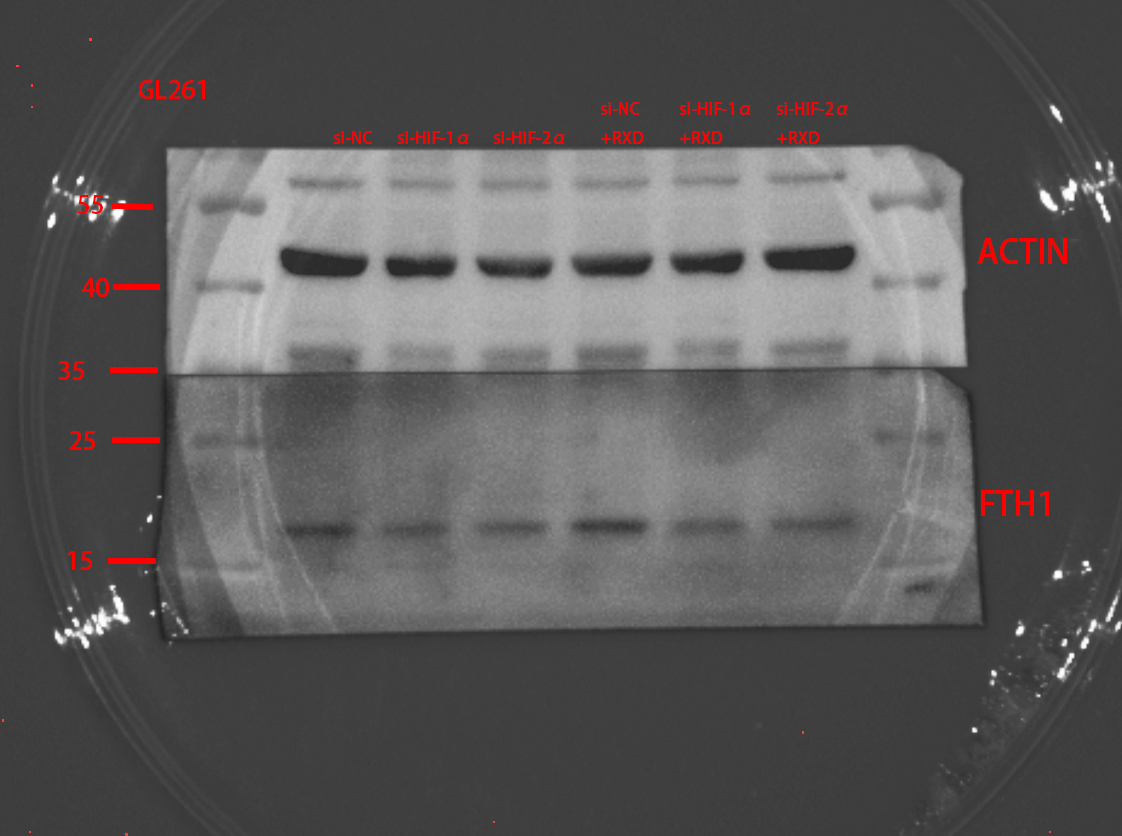


Fig.7B GL261-Gpx4


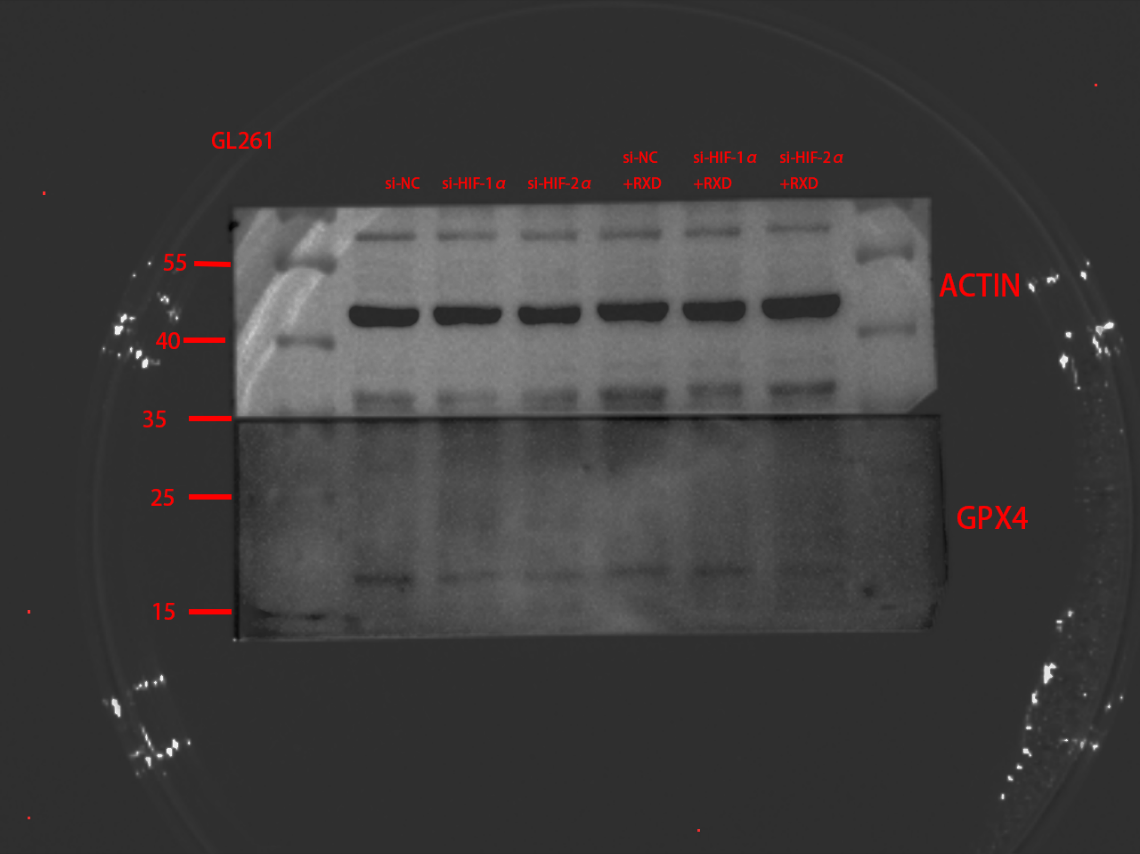


Fig.7B GL261-Hif-1α


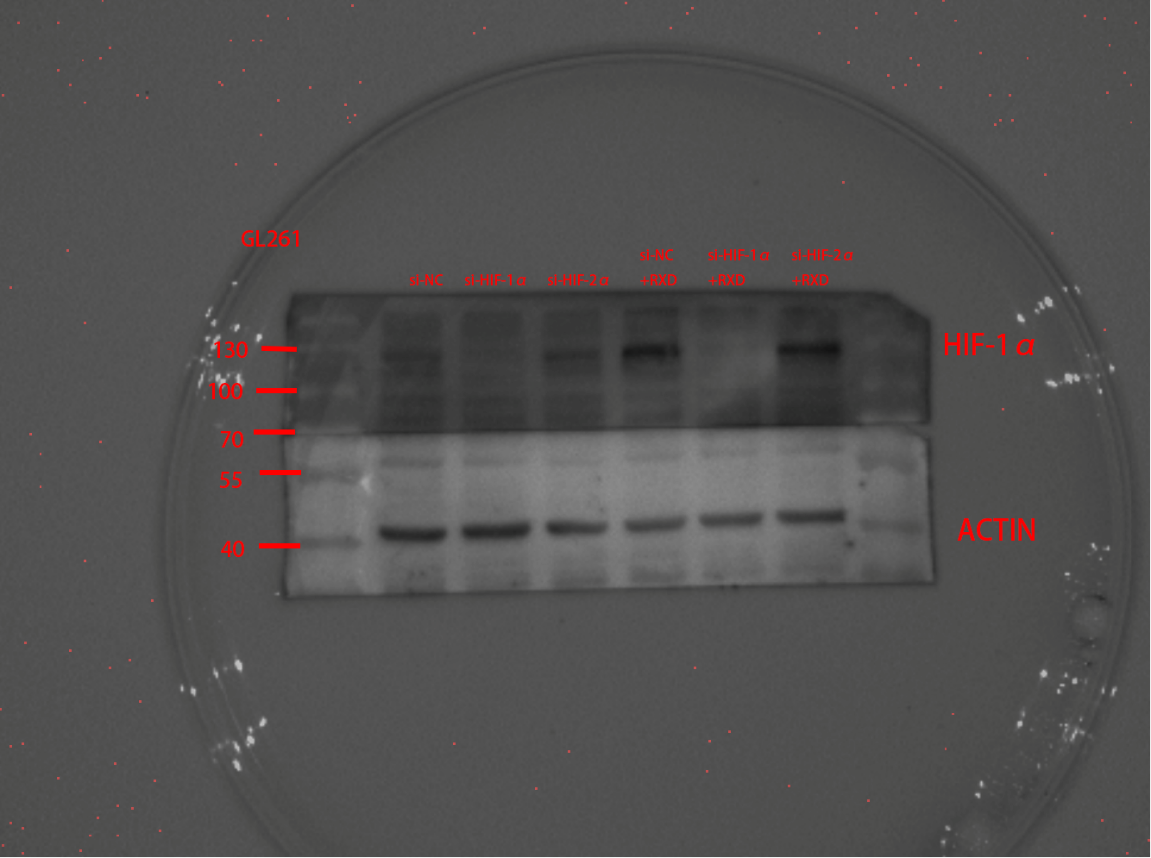


Fig.7B GL261-Hif-2α


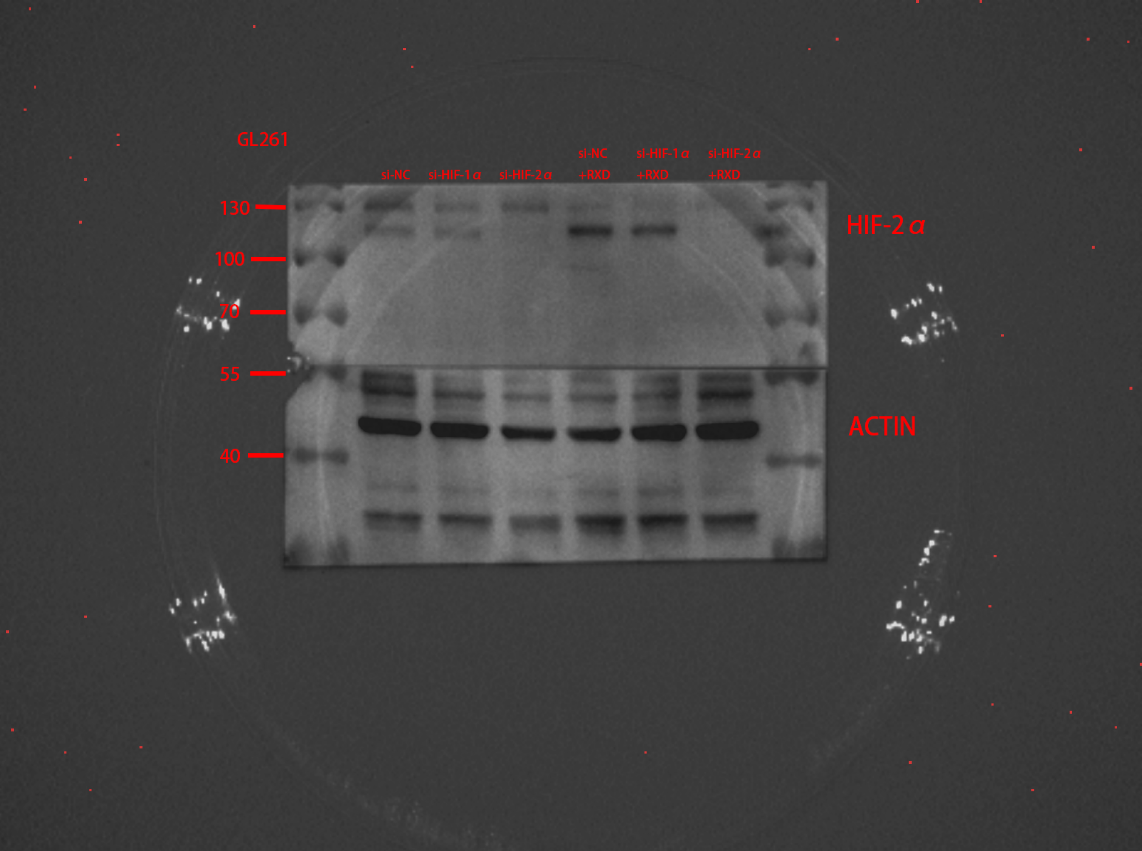


Fig.7B U87-ACTB


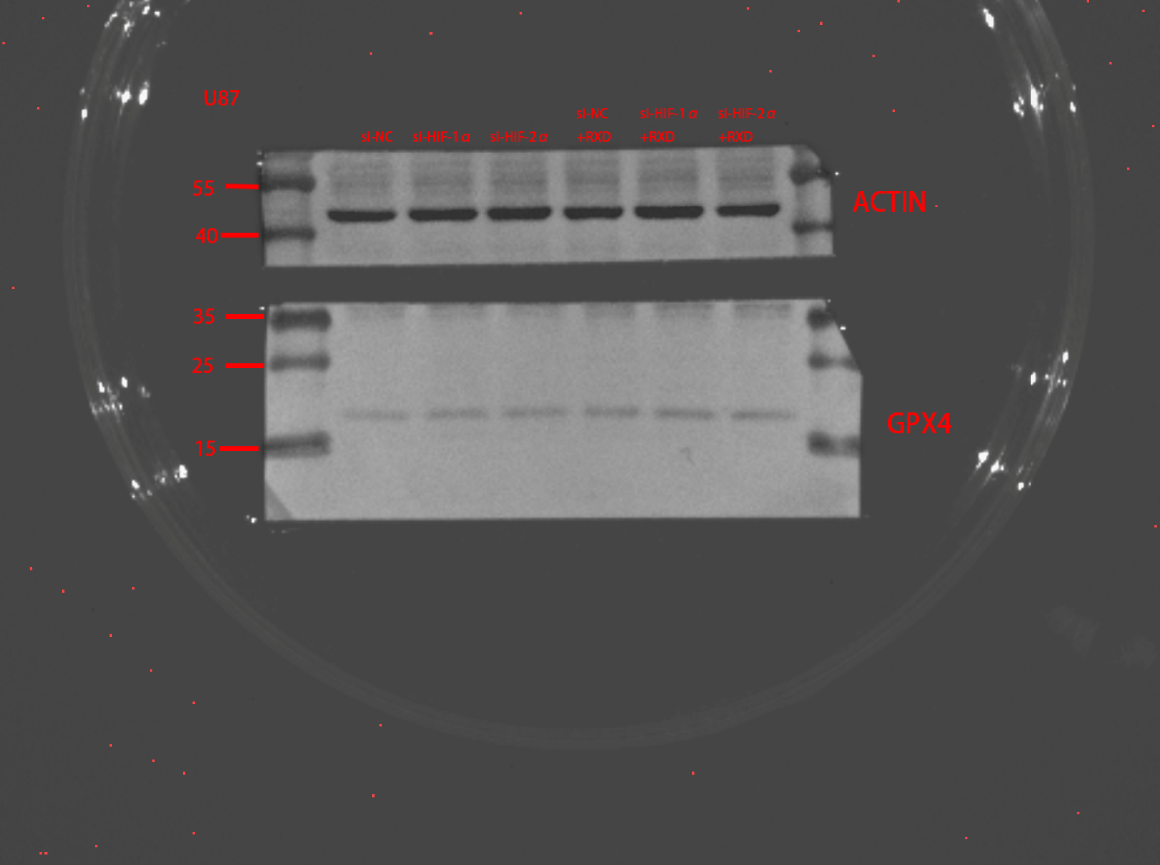


Fig.7B U87-GPX4


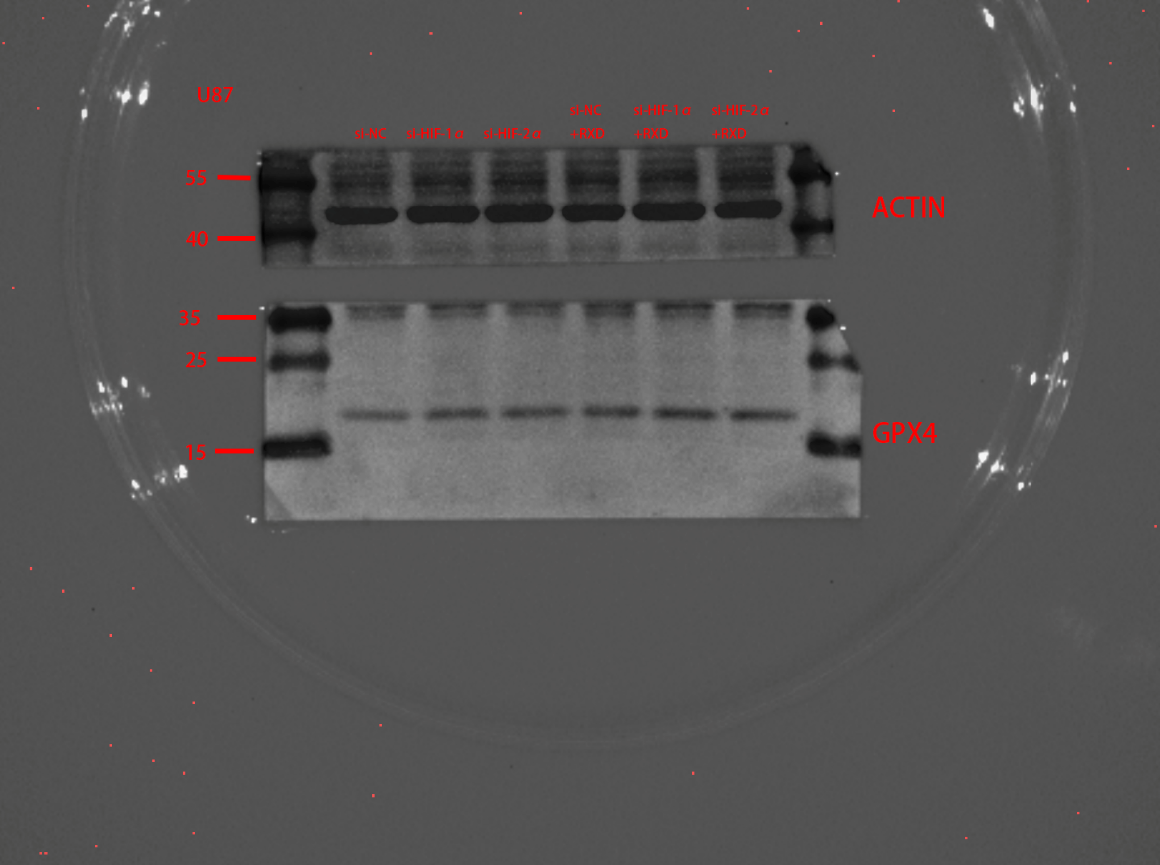


Fig.7B U87-FTH1


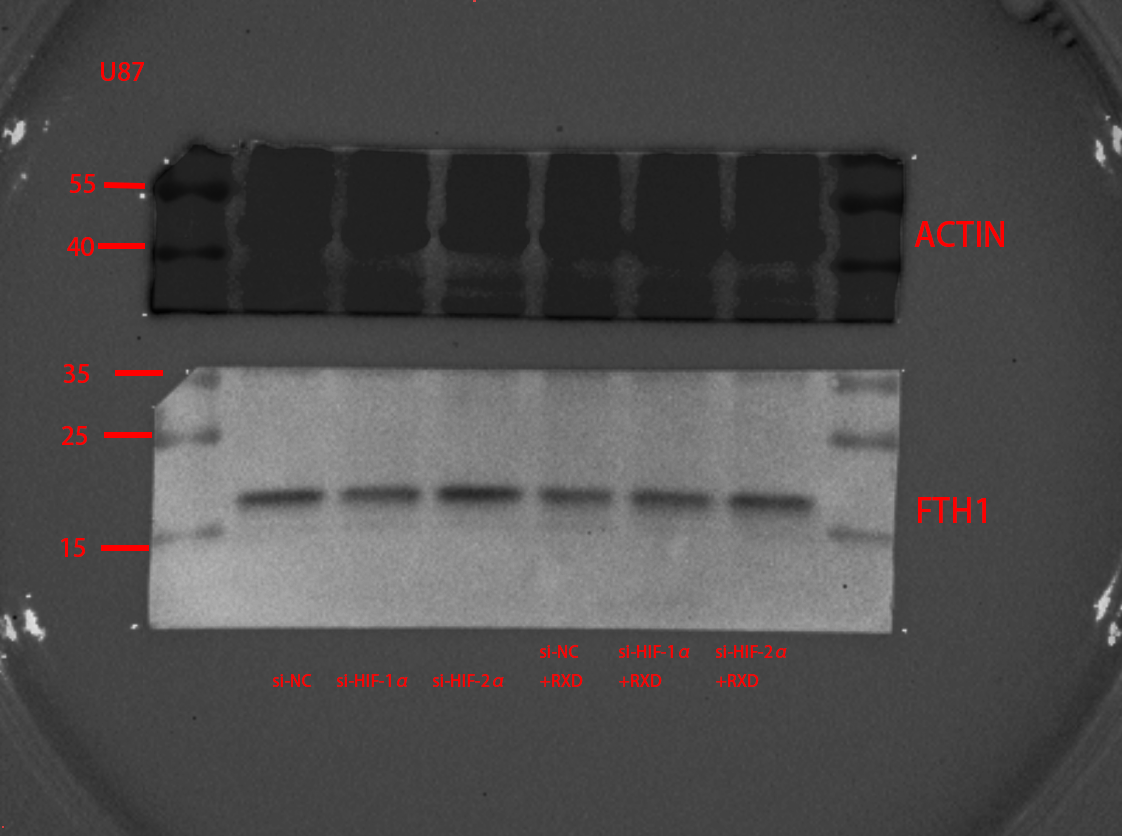


Fig.7B U87-HIF-1α


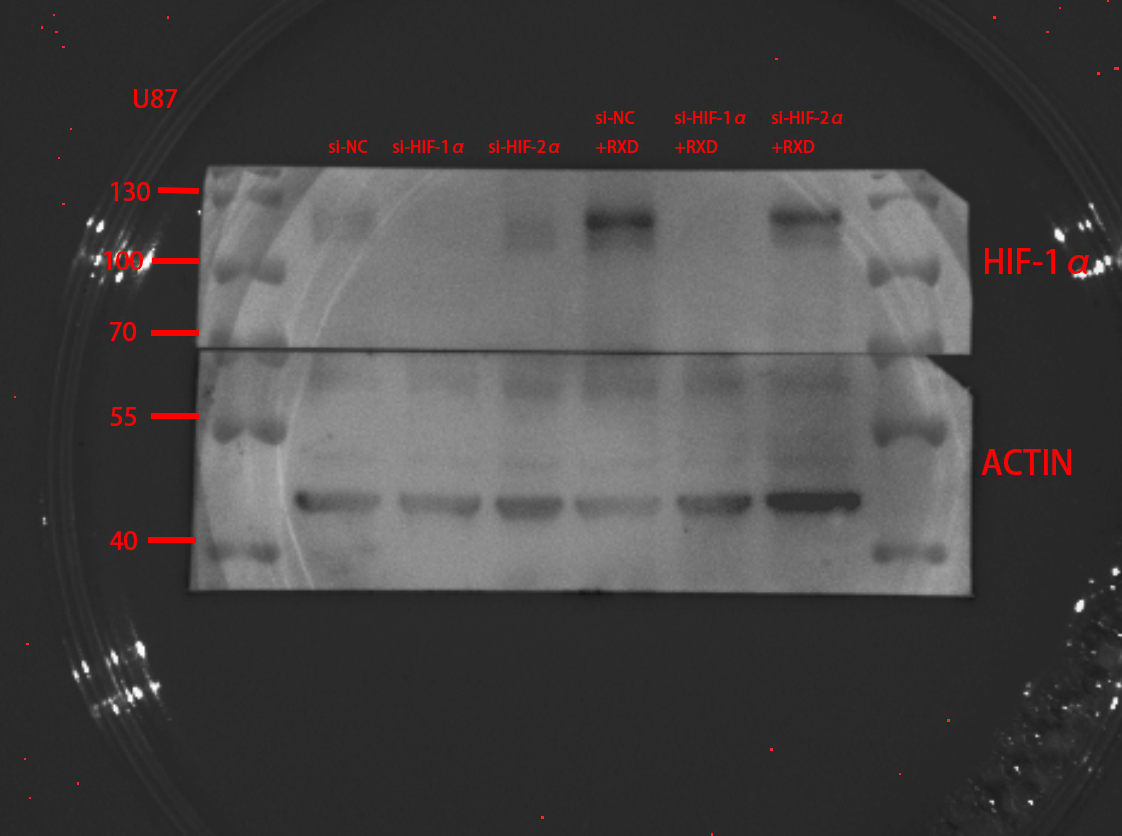


Fig.7B U87-HIF-2α


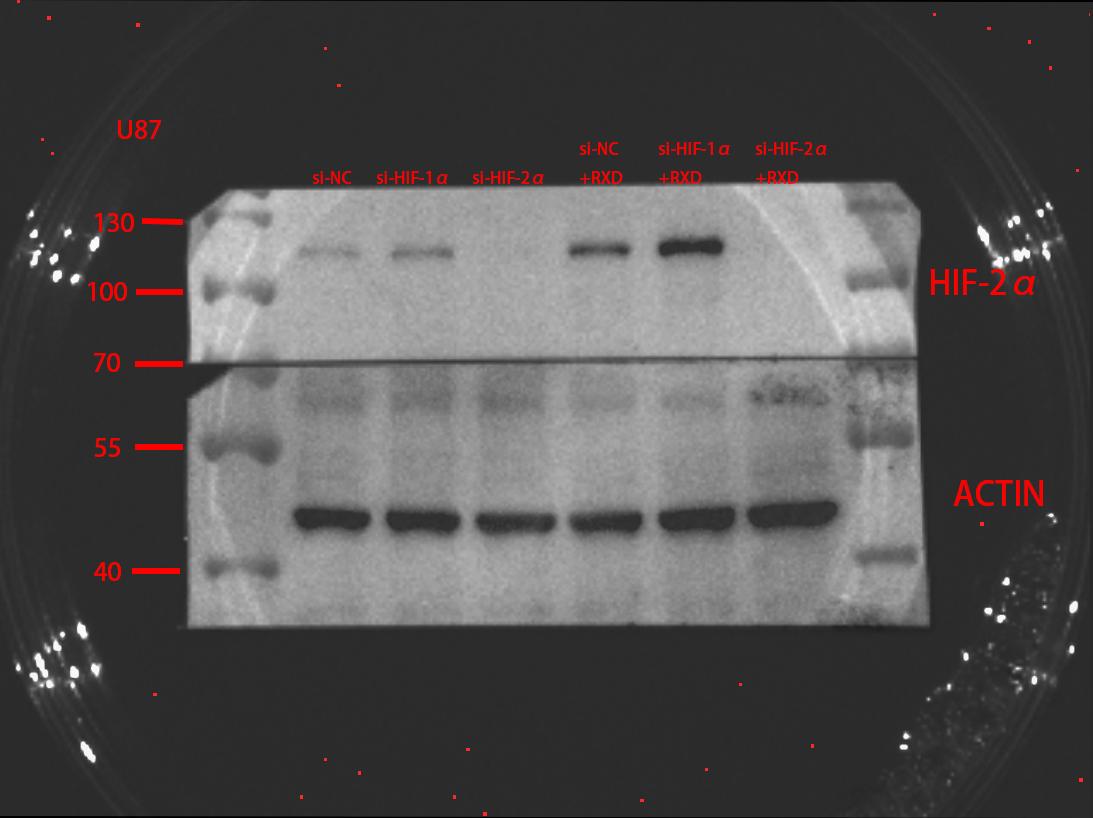

Supplement: Supplementary file 3 — photoes of Raw WB [file 41419_2022_5304_MOESM3_ESM.docx]
